# Mindful Movement Programs in Community and University Preschools: Protocol for a Pilot Randomized Controlled Trial

**DOI:** 10.2196/75731

**Published:** 2025-08-29

**Authors:** Jacinda K Dariotis, Dana A Eldreth, Rachel Jackson-Gordon, Hong Li, Kai-Ning Wang, Chunyuan Xi, Gregory F Lewis, Elizabeth A Shirtcliff

**Affiliations:** 1 Department of Human Development and Family Studies, Family Resiliency Center College of Agricultural, Consumer & Environmental Sciences University of Illinois Urbana-Champaign Urbana, IL United States; 2 Department of Biomedical and Translational Sciences Carle Illinois College of Medicine University of Illinois Urbana-Champaign Urbana, IL United States; 3 Luddy School of Informatics, Computing, and Engineering Indiana University Bloomington Bloomington, IN United States; 4 Department of Psychology College of Arts and Sciences University of Oregon Eugene, OR United States

**Keywords:** mindfulness, mindful movement practices, mindfulness-based intervention, preschool children, early childhood, emotional regulation, stress response, randomized controlled trial, social-emotional learning

## Abstract

**Background:**

Chronic stress and emotional and cognitive regulation difficulties during early childhood are associated with risky behaviors and negative outcomes later in life. As preschoolers are at a developmental stage where their emotional and cognitive regulation skills are still emerging, they often rely on external support to manage their emotions and behaviors. Intervening early promotes healthy development and preventing adverse outcomes. Mindful movement practices (MMPs)—which incorporate physical activities, breathwork, and reflective exercises—show the potential to enhance emotional and behavioral skill regulation. However, the impact of MMPs on cognitive and social-emotional development in young children has not been thoroughly researched, and the underlying mechanisms remain unclear.

**Objective:**

This pilot randomized controlled trial aims to assess the feasibility and acceptability of implementing MMPs in preschool classrooms and explore their effects on preschoolers’ social-emotional, cognitive, and physiological development. The study also focuses on enhancing prosocial behaviors and reducing problem behaviors. This protocol paper outlines the research procedures, sample demographics, and survey data psychometric properties.

**Methods:**

The study was conducted in 3 preschool classrooms (2 intervention and 1 waitlist control). Data were collected among 32 children and 8 teachers. Before the study began, 8 formative focus groups were facilitated with 5 administrators and 33 teachers in total to inform modifications to the program and research design. The intervention group participated in a 16-week program that included neural-based drills, aerobic activities, breathwork, and “feelings circle” exercises, along with classroom and home kits. The waitlist control group received a shortened 8-week program after the intervention groups completed their sessions. Data were collected before the intervention, at the midway point of the intervention, and after the intervention and again 2 months after the intervention. Measures included behavioral tasks, biomarkers (heart rate variability via wearable sensors and hormonal substrates in hair), and surveys from parents and teachers. Implementation fidelity was assessed using quantitative logs, qualitative field notes, and video observations. Postintervention interviews and focus groups were conducted with teachers, parents, and administrators.

**Results:**

Formative focus groups were conducted with 33 teachers and 5 administrators. Behavioral task, biomarker, and survey data were collected from 40 participants, including 32 preschoolers aged 3 to 5 (mean 4.4, SD 0.64) years and 8 teachers with a mean age of 37.9 (SD 11.47) years. Postintervention interviews and focus groups were completed with 7 teachers, 4 administrators, and 8 parents.

**Conclusions:**

This paper describes study procedures. Future publications will present primary outcome findings and evaluate the feasibility and acceptability of this MMP and research design in community-based and university-based preschool settings. Future papers will explore the data to provide preliminary evidence for the program’s effectiveness, mechanisms of effects, and the need for larger randomized controlled trials to optimize early childhood mindfulness interventions.

**International Registered Report Identifier (IRRID):**

DERR1-10.2196/75731

## Introduction

### Background

Chronic stress is an increasing public health concern that often originates in early childhood, especially among vulnerable populations [1,2]. Chronic stress in early childhood—often referred to as early life stress or childhood adversity—is typically measured by adverse experiences such as abuse, neglect, or lack of resources (eg, Smith and Pollak [3]). Persistent stress during early childhood can lead to emotional and cognitive dysregulation, which may later result in risky behaviors during adolescence, including substance abuse, violence, delinquency, and unsafe sexual practices [4-6]. Such outcomes contribute to substantial social and economic burdens in adulthood, manifesting as increased interactions with the criminal justice system, addiction, unemployment, and various health issues [7-9]. Early childhood is a crucial period to develop positive coping strategies via the implementation of evidence-based interventions designed to foster positive trajectories in life.

Preschool years are a pivotal period for the maturation of brain regions associated with motor function, self-regulation, cognition, language, and social competencies [10]. The developmental changes occurring in early childhood present an optimal opportunity for intervention, with preschool environments serving as ideal settings for such initiatives. The traditional social-emotional learning (SEL) programs, such as the well-known Business As Usual program, are standardized programs that have been embedded in the daily routine and classroom curriculum (eg, literacy, math, and circle time), especially focusing on regulating classroom environments and instructional practices [11]. Such a program has been critiqued for limiting responses to individual developmental needs and impeding the integration of comprehensive and inclusive models [12].

Mindful movement practices (MMPs) extend beyond traditional SEL programs by adopting a more developmental and sensitive approach tailored to the unique needs of children. MMPs incorporate movement patterns, attention-focused exercises, breathwork, and static postures, providing effective self-regulation strategies for young children [13-15]. These practices leverage sensory motor systems, cognitive control, and emotional regulation skills to assist children in managing stress and emotional responses [16,17]. Furthermore, the SEL components inherent in MMPs foster positive emotional and behavioral development in children. There are several facets that make this MMP unique from other SEL programs. First, the MMP used in this study adopted a train-the-trainer approach, whereby teachers were trained by program implementers on how to implement the MMP outside of the planned programming schedule. This approach builds teachers’ capacity to implement the program independently. Furthermore, teachers were given calm corner kits to use in the classroom and parents received kits for children to use at home. Using this approach facilitated the reinforcement of learned mindfulness strategies and ensured the consistency of school-home environments to support children’s behavior. By training the teachers and providing calm corner kits, children have more opportunities across settings for SEL.

While most school-based mindfulness research has focused on adolescents, findings show limited effects on externalizing behaviors (eg, aggression) and modest impacts on internalizing behaviors (eg, anxiety) [18-20]. Randomized controlled trials (RCTs) are now emerging that investigate the effects of mindfulness in preschool-aged children [21-24] and the implementation of MMPs in early elementary settings [25]. These studies have shown improvements in self-control, motor function, behavior, executive function, and social-emotional competencies. In addition, MMPs have demonstrated feasibility in urban elementary settings, yielding enhancements in self-control, motor skills, and behavior [25]. Mindfulness practices tailored for preschoolers have also indicated positive outcomes in motor control and social-emotional functioning [21]. These published RCTs show the benefits of mindfulness among preschoolers and school-aged children, but they have not examined which children are most responsive to specific components of MMPs nor have they explored the underlying mechanisms that contribute to the observed changes. Further research is needed to understand the physiological and social-emotional mechanisms underlying MMP effectiveness including identifying which elements of MMPs are most effective for children from diverse racial, sociodemographic, and neurodiverse backgrounds.

Many studies have highlighted the positive impacts of mindfulness-based interventions in children, and there is a growing body of evidence that demonstrates benefits for teachers who participate in mindfulness-based educator training [26-29]. These include minimized teacher burnout, enhanced well-being and social-emotional competencies, cultivation of classroom climates more conducive to learning, teacher-student relationships, classroom management, and self-efficacy [26]. These findings are consistent with the prosocial classroom model, which suggests that teachers’ social-emotional skills and well-being have a positive impact on their relationship with children and classroom management [30]. To our knowledge, no study has examined the effects of mindfulness-based classroom interventions on teacher outcomes. For example, changes in children as a result of mindfulness-based interventions may lead to improved teacher outcomes as mentioned earlier, student-teacher relationships, and attunement in physiological responses.

In response to these gaps, we conducted a pilot RCT to evaluate the hypothesis that MMPs could alter preschoolers’ behavioral difficulties by impacting physiological, social-emotional, and cognitive factors, which, in turn, could affect teacher outcomes. This preliminary evidence will provide support for using these assessments in a future full-scale RCT.

### Objectives

This pilot study was designed to lay the groundwork for a larger RCT using the MMP and building an evidence base to support broader dissemination of an effective, low-cost, highly scalable program. This pilot study has 3 primary aims.

#### Feasibility

This aim focused on assessing the practicality and reception of the intervention and research procedures among preschoolers, teachers, parents, and administrators, including evaluation of the acceptability of data collection methods, such as physiological, behavioral, qualitative, and survey measures. In this study, we draw from the work of Bowen et al [31] and Teresi et al [32], who outline facets of feasibility studies, including factors such as acceptability, implementation, and practicality (among others). These factors may be assessed by consenting to be part of a study, retention, satisfaction, and participation in activities.

#### Effects on Mechanisms and Outcomes

This aim focused on investigating how the implementation of the MMP influences self-regulatory capacities, SEL skills, problem behaviors, and prosocial behaviors, as well as identifying underlying physiological mechanisms of program effects at the midpoint, postintervention time point, and 2-month follow-up. Physiological data were collected after each MMP component, allowing for the evaluation of the effects of sequential implementation on physiological responses.

#### Teacher Effects

This aim focused on evaluating how exposure to MMPs affects teachers’ stress responses due to physiological attunement with children and classroom dynamics, which may subsequently enhance child outcomes.

## Methods

### Setting

Two sites, including 1 community-based site (2 classrooms) and 1 university-based site (1 classroom), participated in the study. These sites were chosen because they serve families from the local community, creating a more heterogeneous sample of children as opposed to focusing on disadvantaged or advantaged populations. Site 1 (community-based preschool program Head Start) had 8 active preschool classrooms, and site 2 (university-based preschool) had 3 active preschool classrooms. Site 1 receives federal funding to provide early childhood education for children from families living below federal poverty guidelines or facing other stressors such as homelessness or disability. Of the enrolled families, 90% were low-income families and 14% received public assistance [33]. Enrollment was determined by need. According to a county-level report for all Head Start sites in the broader study area, approximately 11% of children faced homelessness; 14% were referred for developmental disability or special education; and 32% received Supplemental Nutrition Assistance Program benefits. By comparison, an estimated 13% of the United States receives these benefits (Economic Research Service [34]). Site 2 uses an enrollment model that provides a diverse group of children and families. Using a stratified enrollment model, 25% of enrolled children were children of faculty members, support staff, university students (both undergraduate and graduate), and community families. Approximately 20% to 30% of the children enrolled came from low-income homes (supported by Child Care Assistance Program subsidy vouchers, a grant supporting childcare needs of low-income undergraduate student parents, and a sliding fee scale).

### Participants

A total of 40 participants were enrolled in the RCT part of the study. Participants included 32 preschool children aged 3 to 5 (mean 4.4, SD 0.64) years: 17 (53%) female, 11 (34%) Black, and 4 (12%) Hispanic. Demographics of children were based on parent reports unless those data were missing (n=3, 9%), in which case teacher-reported values were used. In total, 69% (22/32) of the children received the intervention immediately, and 31% (10/32) were assigned to the waitlist control group. Of the 40 participants enrolled across the 3 classrooms, 8 were female teachers with a mean age of 37.9 (SD 11.47) years: 3 (38%) were Black and 4 (50%) were White. A total of 29 parents with a mean age of 33.6 (SD 6.75) years completed the demographic questions: 23 (79%) female, 12 (41%) Black, and 2 (7%) Hispanic. Baseline demographics by intervention group for children and parents are presented in Tables 1 and 2.

**Table 1:** Demographic characteristics of children (N=32).

Characteristics		Full sample	Intervention (n=22)	Control (n=10)
Age (y), mean (SD)		4.4 (0.64)	4.6 (0.50)	3.9 (0.72)
**Sex n (%)**				
	Male	12 (55)	—^a^	—
	Female	17 (53)	10 (45)	7 (70)
**Ethnicity, n (%)**				
	Hispanic or Latinx	—	—	—
	Not Hispanic or Latinx	28 (88)	21 (95)	7 (70)
**Race, n (%)**				
	Asian, Native Hawaiian, or Other Pacific Islander	6 (19)	5 (23)	—
	Black or African American	11 (34)	6 (27)	5 (50)
	White	15 (47)	11 (50)	4 (40)
**Language, n (%)**				
	Native English speaker	20 (62)	13 (59)	7 (70)
**Disability, n (%)**				
	None	27 (84)	17 (77)	10 (100)
	One or more disabilities	5 (16)	5 (23)	0 (0)

^a^Samples sizes of <5 participants.

**Table 2:** Demographic characteristics of parents (N=29).

Characteristics			Full sample		Intervention (n=19)		Control (n=10)
Age (y), mean (SD)			33.6 (6.75)		34.8 (5.64)		30.9 (8.36)
Number of children, mean (SD)			2.1 (1.11)		2.0 (0.77)		2.4 (1.57)
**Sex, n (%)**							
	Male	6 (21)		6 (32)		—^a^	
	Female	23 (79)		13 (68)		10 (100)	
**Ethnicity, n (%)**							
	Hispanic or Latinx	—		—		—	
	Not Hispanic or Latinx	27 (93)		19 (100)		8 (80)	
**Race, n (%)**							
	Asian, Native Hawaiian, Other Pacific Islander	—		—		—	
	Black or African American	12 (41)		5 (26)		7 (70)	
	White	13 (45)		10 (53)		—	
**Education level, n (%)**							
	High school degree or GED^b^	9 (31)		5 (26)		—	
	Some college but no degree	6 (21)		—		—	
	Associate or bachelor degree	7 (24)		—		—	
	Graduate degree	7 (24)		7 (37)		—	
**Marital status, n (%)**							
	Single, living alone	6 (21)		—		—	
	Living with a romantic partner	—		—		—	
	Married	16 (55)		14 (74)		—	
	Separated or divorced	—		—		—	
**Employment status, n (%)**							
	Employed for wages or self-employed	23 (79)		13 (68)		10 (100)	
	Unemployed	6 (21)		6 (32)		—	
**Income range (US $), n (%)**							
	≤$26,000	8 (28)		6 (32)		—	
	$27,000 to $49,000	12 (41)		5 (26)		7 (70)	
	≥$50,000	7 (24)		7 (37)		—	

^a^Samples sizes of <5 participants, except for reported missingness.

^b^GED: General Educational Development.

### Procedures

This pilot study consisted of 8 formative focus groups with administrators and teachers who aimed to inform modifications to the study design before data collection and implementation. The RCT took place from January 2024 through August 2024 and consisted of multiple components (detailed below).

#### Formative Interviews and Focus Groups

In fall 2023, 6 focus groups were conducted with 33 teachers and 2 focus groups were conducted with 5 administrators. These formative focus groups were conducted at 3 Head Start locations and the university preschool to gauge teacher and administrator interest in participating in research studies and a proposed intervention study. All 33 teachers expressed positive sentiments about the program being implemented in classrooms; however, because this was a pilot study, we could only implement the program in 3 classrooms across 2 sites. Classrooms were selected based on the children served (those aged 3 to 5 years), teacher buy-in, and administrator feedback on the feasibility of implementation (eg, full-day classrooms vs half-day classrooms). The future full-scale RCT will randomize classrooms to conditions. The research team followed up with teachers in 3 identified classrooms and confirmed their willingness to participate in the research study before classroom selection was finalized.

#### Randomization Procedures

At site 1, a total of 2 classrooms were randomly assigned to receive the 16-week intervention or serve as the waitlist control group. Because randomization was only needed for 2 classrooms at site 1, we used a manual procedure by writing classroom numbers on pieces of paper, which were then folded and mixed, and one was drawn to assign the intervention group. The one classroom at site 2 was assigned to receive the 16-week intervention. A 2:1 ratio between the intervention and control groups was chosen to maximize the number of children receiving the intervention while maintaining a comparison group for evaluating the effectiveness of the program.

#### The Mindful Movement Program

The MMP combined movement practices (Mission2Move [35]) and positive behavioral intervention and supports (PBIS; Calm Caterpillar [36]) into one progressive program, referred to as M2M-CC (Mission2Move and Calm Caterpillar) for short. M2M-CC integrated evidence-based movement (Z-Health [37]) and meditation (Mindful Schools [38,39]) strategies. It was an SEL program that provided a mindful movement curriculum and tools to support classrooms and child development. M2M-CC practices included (1) neural-based drills, (2) aerobic activity, (3) breathwork and guided meditation, and (4) “feelings circle” or reflective practices. Positive behavioral intervention occurred with the use of visual and tactile aids, and support was provided through classroom or home kits that children, parents, and teachers could use in times when children needed assistance in regulating their emotions and behaviors. The movement (neural-based drills and aerobic activity) and mindfulness (breathwork and guided meditation, as well as reflective practices and “feelings circle”) practices worked in conjunction with each other to teach children how physical health is tied to social-emotional well-being and goal attainment [40]. Teachers and parents received a calm corner kit (classroom or home kits) near the end of the program to help children practice self-regulation techniques at home and in the classroom when direct instruction was not occurring.

#### Program Implementation

For the study, the program was delivered twice per week in classrooms receiving the intervention. One weekly session was a 15-minute mindful movement class and the other session was a 5- to 7-minute refresher or booster class. These classes were taught over 16 weeks (immediate intervention group) and 8 weeks (waitlist control). Study implementers were trained and supported by the MMP developer, one person led the implementation of the program to create consistency across sites, and an established curriculum was used to maintain standardization of the program. Before programming began, teachers received professional development training. During programming, teachers were present for direct instruction by study implementers so that they could learn the MMPs for use outside of planned programming and booster sessions each week and for future classroom implementation.

A 4-part sequence of program components was administered over time. First, children warmed up with “neural-based drills.” Drills used specific movements to train the nervous system in how body parts were supposed to move. These increased children’s body awareness in movement, location, and action (ie, proprioception). Neural drills included movements such as hip circles or ankle tilts. In addition to neural drills, children were led in aerobic movement–focused activities (eg, dance and running in place). Second, children learned a variety of “simple breathing practices” that used different techniques for varied benefits. For example, children learned hand breathing—inhaling and tracing up a finger, then exhaling and tracing down a finger—to support self-calming. Third, children engaged in a reflective practice known as “feeling circles,” where they talked about the meaning of different feelings and shared when they experienced the feeling. We used a “talking stick” approach whereby each child had the opportunity to share and be heard when they held the stick. Finally, classrooms and parents were provided a kit of tools for creating a calming corner as another component of PBIS to reinforce skills taught in the program.

As presented in Table 3 (based on a 15-minute mindful movement class), mindfulness program components were sequentially introduced every 4 weeks after the first component. For example, during a 16-week implementation, components were added every 4 weeks (weeks 1-4, 5-8, 9-12, and 13-16). In an 8-week-long administration, program components were added every 2 weeks. Movement was first introduced, followed by breathwork and meditation, then feeling circles or reflection, and finally the calm corner (PBIS). This allowed for assessing program component effects on physiological (ie, respiratory sinus arrhythmia and vagal efficiency) and SEL mechanisms.

**Table 3:** Programming component description, impact, and implementation details.

Component	Description		Hypothesized impact	Timing		Weeks		Modality		Location		Method	
Neural drills	Activate the brain through specific body movements (eg, hip and arm circles)		Learn body awareness and inhibitory control	5 min		1-16		Movement		School		Direct instruction	
Aerobic activities	Elevate heart rate via different activities and movement sequences	Reduce overall stress and cortisol			10 min		1-16		Movement		School		Direct instruction
Breathwork and meditation	Practice breathing techniques (eg, hand, belly, lion’s breath, and body scan meditation)	Practice control over emotions			2-3 min		5-16		Mindfulness		School		Direct instruction
Feelings circles	Label feelings and share personal examples with the circle and emotion recognition	Practice reflection and learn empathy and interpersonal connection			2-3 min		9-16		Mindful		School		Direct instruction
Calming corner	Down-regulation strategies for children included a classroom kit and a home kit for parents, a quiet space with reduced stimuli, visual aids for self-calming, and tactile weighted toy for soothing and counting	Practice self-calming skills; removed from environmental stimuli (eg, sounds, people)			Varies		13 to ≥16		Positive behavioral intervention and supports		School and home		Child and adult self- guided

#### Waitlist Control Procedures

For consistency of exposure to research team personnel, both the immediate intervention and waitlist classrooms were visited by the implementation team twice weekly for 20 minutes per week. For the waitlist classroom, implementers interacted with children during typical classroom activities (eg, reading and playing with sensory tables).

Following the 16 weeks of programming and postassessment time point, the waitlist group received 8 weeks of the MMP with each program component sequentially added every 2 weeks. This condition allowed for testing of shorter program dosage effects than longer ones.

#### Data Collection Timeline

**Figure 1:**
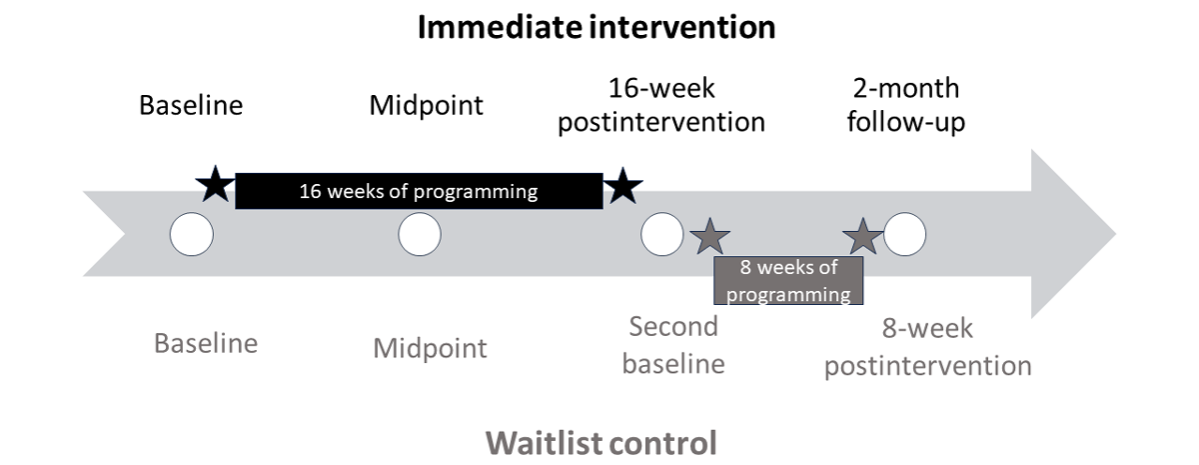
Overview of data collection time points for the experimental group.

Data were collected at 4 time points: baseline (preintervention; all classrooms), midintervention time point (8 weeks into programming; all classrooms), postintervention time point (immediately following the 16-week intervention or waitlist period; all classrooms), and 2-month follow-up (2 months after the intervention; 1 intervention classroom). In the waitlist control group, the postintervention time point also served as their second baseline and the 2-month follow-up served as their postintervention time point (immediately after the 8-week intervention). The data collection time points are illustrated in Figure 1 for the intervention (black) and waitlist control (gray) groups. At each time point, survey data were collected electronically (in person or online) or via paper and pencil, whichever was feasible and preferred by participants. Behavioral assessments were administered at all time points except for the midintervention time point.

### Outcome Measures

#### Behavioral Assessments

The pencil tapping task assessed working memory and inhibitory control [41,42]. In this task, both the evaluator and the child held a pencil in their dominant hand. The child was instructed to tap the pencil twice when the evaluator tapped once and to tap once when the evaluator tapped twice. This required the child to hold the rules in working memory while inhibiting the natural tendency to mirror the evaluator’s tapping pattern. Up to 6 practice trials were used before the official assessment began. During the practice trials, the evaluator provided feedback on correct and missed trials to ensure understanding of the task. Following the practice trials, 16 scored trials were administered without feedback. The total number of correct responses ranges from 0 to 16.

The gift delay task assessed self-regulation [43]. In this study, it also served as a stress task to assess child coping and stress response (eg, behavioral and physiological response via heart rate variability [HRV], respiratory sinus arrhythmia, and vagal efficiency). During this task, the child sat at a table while the experimenter presented a gift bag containing a prize, explaining that the bag needed a bow before the child could open it. The experimenter placed the bag in the center of the table and instructed the child to remain seated and not touch the bag until she returned. After a 3-minute delay, the experimenter returned, placed the bow on the bag, and gave the child the gift, regardless of whether they followed the instructions. Scoring was based on the child’s ability to resist the temptation to touch the gift bag during the waiting period. Children were given a score of 1 if they took the gift from the bag, 2 if they put their hand in the bag, 3 if they peeked in the bag, 4 if they touched the bag, and 5 if they followed all instructions. Scores ranged from 1 to 5. Time spent out of the seat was also calculated and categorized into 4 distinct levels: 1 for <30 seconds in the seat to 4 for >2 minutes in the seat. A final seat score was computed by assigning an additional half point for each minute a child spent out of the seat within arm’s reach of the table for those with scores of 1 or 2. The task was video recorded using a discreet camera for later review and double scoring by a second researcher.

The tower task assessed turn-taking and inhibition [43]. During this task, the child sat at a table with the experimenter seated across from them. The child was given a practice trial where they were instructed that they would be building a tower together by taking turns. First, the experimenter placed a block on the table and instructed the child to place the next block on top. The practice trial ended after the tower was 6 blocks tall. During the practice trial, they were reminded to take turns if they were not taking turns. The actual trial consisted of 12 blocks and the experimenter told the child that they would build a really tall tower together and the child could go first. Children were scored on 2 characteristics. First, on turn-taking: 0 for no turns (children built the tower by themselves), 1 for partial turn-taking, or 2 for full turn-taking. A second turn-taking score was calculated based on the proportion of blocks placed by the experimenter (multiplied by 10) relative to the total number of blocks placed. The child received the highest score if they alternated placing blocks equally with the experimenter. A penalty of 5 points was applied if the child knocked the tower over, while an additional 5 points were awarded if the child gently removed blocks from the tower during cleanup. Children were given 2 minutes to clean up the tower and were scored 2 if they cleaned up the towers on the first try, 1 if they needed follow-up after 1 minute, or 0 if they did not clean up the tower.

The go/no-go task assessed inhibitory control in young children [44-48]. In our study, we used an app-based version of the go/no-go task on an Apple iPad. Children were tasked with catching fish (go trials) and avoiding sharks (no-go trials) as they moved across the screen [49]. To enhance the dominant response and increase the difficulty of inhibiting responses during the “no-go” trials, 80% (n=60) of the trials were “go” trials and 20% (n=15) were “no-go” trials. Before starting, children received instructions on both trial types and practiced 5 trials of each. This was followed by a mixed block of 10 practice trials (with 80% being “go” trials). A brief review of the instructions was provided before the task began. The main task consisted of 75 test stimuli evenly distributed across 3 blocks. The stimuli were presented in pseudorandom order, ensuring that no block began with a “no-go” trial and that no more than 2 consecutive “no-go” trials occurred. A 1000-millisecond interval was maintained between stimuli. Scores were calculated by first excluding trials with response times <300 millisecond, as these were considered non–task related. Next, blocks were removed if the child demonstrated nonresponsiveness (ie, go trials with <20% accuracy or no-go trials with >80% accuracy) or indiscriminate responding (ie, go trials with >20% accuracy and no-go trials with <20%). The impulse control score, which reflects the ability to withhold a response in the presence of a strong, automatic tendency to respond, was calculated by multiplying the percentage of correct “go” responses by the percentage of correct “no-go” responses.

#### Biomarkers

##### Overview

Data were collected to assess potential physiological mechanisms driving the intervention effects. These mechanisms were evaluated across several biological systems: the autonomic nervous system, the hypothalamic-pituitary-adrenal (HPA) axis, and the hypothalamic-pituitary-gonadal (HPG) axis. These systems were chosen because they play key roles in self-regulation and the body’s response to stress and challenge or threat [50].

##### HRV Measurement

HRV assesses acute stress response and reflects the balance between the sympathetic nervous system and parasympathetic nervous system, both of which are integral to maintaining homeostasis [51]. HRV is a well-established biomarker of an individual’s ability to regulate and recover from stress [52].

For teachers and children who opted into this component of data collection, HRV data were collected at multiple time points: before the intervention, every 4 weeks during implementation (with each new module), and at postintervention and 2-month follow-up assessments. To collect HRV data, trained research assistants attached Firstbeat Body Guard sensors (Firstbeat Technologies Ltd [53]) to the participants—both children and teachers—by placing them beneath the right collarbone and connecting the sensor cable to the rib cage on the opposite side [54]. If a participant had sensitivities to its placement on the front of the body, electrodes were placed in these equivalent locations on the back. The sensors were removed once data collection was complete.

##### HRV Collected During Implementation

For children, the baseline HRV was measured while they were sitting still or engaged in low-physical movement during class activities for approximately 3 to 5 minutes. During each program module, HRV was measured in both the active and recovery states, with sensors worn for around 20 minutes during the program implementation days. Teachers wore the sensors at the same time points as the children, both before and after the program, during normal classroom activities, and for the same amount of time as the children during the program.

##### HRV Collected During Behavioral Tasks

Children wore Firstbeat sensors while completing all the tasks, especially the gift delay task that was designed to induce moderate psychological stress. This task, commonly used in research to provoke a physiological stress response [55,56], was assessed via data collected at preintervention, postintervention, and 2-month follow-up time points. The task sequence involved 3 phases. First, there was a 3-minute baseline phase, where children colored quietly to establish a baseline HRV measurement. This was followed by a 3-minute gift delay task, which was intended to evoke the stress response. Finally, there was a 3-minute recovery phase, where children had time to recover after the stressor. Throughout the paradigm, heart rate and pulse wave activity were continuously monitored to assess changes in vagal tone, allowing for the measurement of both reactivity (the body’s immediate response to stress) and recovery (how well the body returns to baseline following stress). This procedure of baseline, task, and recover was followed for the pencil tapping, tower building, and go/no-go task as well, even though they were not hypothesized to elicit the same stress response as the gift delay task.

##### Hormone Measurements

Hair was collected to measure cortisol, testosterone, and dehydroepiandrosterone (DHEA) levels to capture chronic stress and threat-challenge exposure. Cortisol, a hormone secreted by the HPA axis, is known to rise in response to stress [57]. Testosterone, produced by the HPG axis, also increases in response to challenging situations [58,59]. DHEA is closely linked to both cortisol and testosterone and is needed to tease out the unique coupling effect. DHEA is produced adrenally in both the gonads and the brain. DHEA and its sulfated analog are the most abundant hormones in the body, and as an adrenal androgen, DHEA operates as an end-product of both HPA and HPG axes [60]. By examining the coupling of HPA and HPG axes activity, both independently and together, we can better predict the risk of negative health outcomes related to stress [61]. In addition, exploring HPA and HPG coupling broadens the understanding of stress from the traditional “fight-or-flight” response to a more nuanced “threat-challenge” framework, which can apply to a wider range of real-world stressors [58,62].

Hair samples were collected from both children and teachers who consented to participate in this component of the study. These samples were used to assess cortisol, DHEA, and testosterone levels, as hair is highly sensitive to hormonal changes. Hair sampling is more feasible than saliva collection in preschool-aged children, and hormone measurement is more reliable in hair than fingernail clippings. Hair samples were collected at 3 time points: preintervention, postintervention, and 2-month follow-up time points. A 1-cm section of hair reflects approximately 1 month of growth. Therefore, with hair lengths of several centimeters, we were able to assess the change of time in hormonal activity, with the closest hair to the scalp representing the most recent hormone accumulation [63,64].

Trained research assistants cut a small section of hair, containing around 50 to 100 strands, from the posterior vertex of the participant’s scalp following standardized hair sampling procedures [63]. If necessary, multiple small samples from the same region were collected to ensure that there was sufficient hair for analysis. The samples were preserved in foil and envelopes and labeled with the date and participant ID. Family members were also given the option to provide hair samples on site or follow instructions to cut them at home. The research team made efforts to accommodate different preferences for hair sample collection.

#### Surveys

##### Overview

Surveys containing standardized questionnaires were completed by parents and teachers either via paper and pencil or electronically via Qualtrics (Qualtrics International Inc), depending on preference and availability. Lead and assistant teachers completed surveys about themselves (self-report), and lead teachers completed surveys about each child in their classroom. Parents completed surveys about their children and a self-report survey about their perceived stress. Table 4 provides the list of survey questionnaires and scales collected at each time point and by respondents. Published scoring guidelines were used for all standard scales and questionnaires.

**Table 4:** List of survey assessments and time points collected during the study.

Report type and category		Measure	Respondent	Time point
**Report on children**				
	Sociodemographic	Age, sex, race or ethnicity, disabilities, home language, and language proficiency	Parent and teacher	Before intervention
	Adversity	Adverse Childhood Experiences Questionnaire	Parent	Before intervention
	Social-emotional behavior	Children’s Behavior Questionnaire– very short form Strengths and Difficulties Questionnaire Open-ended questions^a^	Parent and teacher	Before intervention, after intervention, and follow-up
	Relationship and implicit bias	Student-Teacher Relationship Scale–short form Preschool expulsion risk measure Open-ended questionsa	Teacher	Before intervention, after intervention, and follow-up
**Self-report**				
	Sociodemographic	Age, sex, race or ethnicity, highest level of education achieved, household income, marital status, employment status, number of kids, and teachers’ role^b^	Parent and teacher	Before intervention
	Stress level	Perceived Stress Scale	Parent and teacher	Before intervention, after intervention, and follow-up
	Teachers’ changes	Teacher SEL^c^ Beliefs Scale Mindfulness in Teaching Scale	Teacher	Before intervention, after intervention, and follow-up

^a^These were also administered at the midintervention time point.

^b^Data collected only from teachers at follow-up.

^c^SEL: social-emotional learning.

##### Teacher Reports on the Children

Children who had parental consent were rated by lead teachers on preschool expulsion risk, student-teacher relationship quality, behaviors, and strengths and difficulties.

The Preschool Expulsion Risk Measure was used to measure implicit bias [65]. This 12-item scale included 4 subscales with 3 items each: classroom disruption, fear of accountability, hopelessness, and teacher stress. Items were rated on a 5-point Likert scale ranging from 1 (strongly disagree) to 5 (strongly agree), and mean scores were calculated. Higher scores represented a greater risk of expulsion.

The Student-Teacher Relationship Scale–short form was used to assess teachers’ perceived relationship quality with individual students [66]. This 15-item scale included 2 subscales: closeness (8 items) and conflict (7 items). Items were scored on a 5-point Likert scale ranging from 1 (definitely does not apply) to 5 (definitely applies). Mean scores were calculated with higher scores indicating more quality.

Open-ended questions were included in surveys at each time point. The teacher surveys included questions regarding expectations for each child in the MMP, what makes the job of teaching the child more or less enjoyable, and changes in the child’s behavior or typical behavior at school in general and during stressful situations over the course of the intervention. The teacher self-report surveys included questions related to feedback on having the research team visit their classrooms. Teachers were given the opportunity to comment about additional information they liked to share at the end of each survey.

##### Teacher and Parent Reports on the Children

The Children’s Behavior Questionnaire–very short form was administered to evaluate child temperament [67,68]. This 36-item questionnaire contained 3 subscales: surgency or extraversion, negative affectivity, and effortful control. The surgency or extraversion subscale included 12 items about a child’s sociability and activity level. Negative affectivity was a 12-item subscale that assessed the frequency of negative emotions, such as frustration. Effortful control was a 12-item subscale that focused on a child’s ability to regulate attention and manage impulses. Respondents were asked to read each statement and indicate the child’s reaction to that situation. Items were rated on a 7-point Likert scale ranging from 1 (extremely untrue) to 7 (extremely true). If a respondent had not observed the child in the situation, the response was left blank. Mean scores for each subscale were calculated with higher scores representing poor temperament.

The Strengths and Difficulties Questionnaire was a 25-item scale used to measure behavioral and emotional strengths and difficulties in children and young people [69]. This scale was divided into 5 subscales of 5 items each: hyperactivity, emotional symptoms, conduct problems, peer problems, and prosocial behavior. Each item was scored on a 3-point Likert scale ranging from 0 (not true) to 2 (certainly true). Sum scores were calculated for each of the 5 scales. A composite total difficulties score was generated by summing the scores for hyperactivity, emotional symptoms, conduct problems, and peer problems. Higher scores for hyperactivity, emotional symptoms, conduct problems, peer problems, and total difficulties denoted worse behavior, whereas higher prosocial scores represented better behavior.

##### Parent Reports on the Children

The Adverse Childhood Experiences Questionnaire was a 10-item measure used to identify traumatic events experienced during childhood, specifically involving abuse, neglect, and household dysfunction [70]. Each item prompted respondents to indicate whether specific events occurred in their child’s life, with answers scored as 1 (yes) and 0 (no). A total Adverse Childhood Experiences Questionnaire score was generated by summing the responses across all items. For this study, only 8 items were used; items regarding reportable events were removed. The minimum score was 0 and the maximum was 8, with a higher score indicating greater exposure to adverse childhood experiences.

The parent surveys about their child included open-ended questions regarding what makes parenting them more or less enjoyable and changes in the child’s behavior or typical behavior at home in general and during stressful situations that occurred over the course of the intervention. In the middle of programming, parents with children in the intervention group were asked whether their child shared anything with them about the program. At the end of classroom programming, parents were asked questions about using the calming corner kit at home. Parents were given the opportunity to comment about additional information they liked to share at the end of each survey.

##### Teacher and Parent Self-Reports

The Perceived Stress Scale was used to assess how different situations affect feelings and perceived stress over the past month in both teachers and parents [71]. This scale includes 10 items rated on a 5-point Likert scale ranging from 0 (never) to 4 (very often). Scores range from 0 to 40 with higher scores representing greater perceived stress.

The Mindfulness in Teaching Scale was used to assess teachers in the context of their teacher activities [72]. This 16-item scale is divided into 2 subscales: 8-item Intrapersonal Teacher Mindfulness and 8-item Interpersonal Teacher Mindfulness. Items are rated using a 5-point Likert scale, from 1 (never true) to 5 (always true). A total of 8 items were reverse coded such that subscales and total mindfulness score calculated sums had higher scores, indicating a greater level of mindful teaching.

The Teacher Social-Emotional Learning Beliefs Scale was a 12-item scale that assessed teachers’ beliefs about SEL [73,74]. This measure was divided into 3 subscales, each with 4 items: comfort with teaching SEL, commitment to learning about SEL, and school culture surrounding SEL. Respondents were asked to read each item and rate their agreement with each item for the past 4 weeks on a 5-point Likert scale from 1 (strongly disagree) to 5 (strongly agree). Composite scores for each subscale were generated by summing each item’s scores, with scores ranging from 4 to 20.

#### Postintervention Interviews and Focus Groups

Between June and August 2024, qualitative data were collected through interviews and focus groups with teachers, parents, and administrators. These interviews and focus groups gathered insights into teachers’ and parents’ perceptions of the program’s effectiveness, challenges encountered, and suggestions for improving the program. In the intervention group, 5 teachers completed interviews, 4 parents participated in 2 focus groups and 1 parent completed an interview. A total of 4 interviews were conducted with administrators. In the control group, there were 2 interviews with teachers and 3 with parents after the intervention.

The protocols for the qualitative data collection were adapted from protocols used in other qualitative studies of mindfulness programs [75,76]. Modifications were made by the project team to capture a wide range of insights. The protocols for each set of reporters began with questions or probes about experiences with the program (eg, “Describe your experiences working with implementers.”); perceived changes in participants (eg, “Since the program began, what changes, if any, have you observed?”); and experiences with research components (eg, What was your experience with these data collection components?). The wording was modified based on whether the interviewee was a parent, teacher, or administrator. Protocol questions were informed by team members’ foundations in implementation science, addressing broad constructs such as barriers and facilitators of implementation, as well as implementation outcomes such as acceptability or sustainability, more specifically [77,78]. For example, 1 question was, “Thinking about the next academic year, what are your hopes for the programming to continue in your school? What supports will help it continue?”

#### Program Implementation Measures

Several strategies were used to assess implementation fidelity, acceptability, feasibility, and sustainability. In addition to the qualitative interviews, focus groups, and open-ended survey questions about program components (described in the Surveys section and the Postintervention Interviews and Focus Groups section), implementation was documented through (1) implementer-reported fidelity assessment forms, (2) teacher-reported activity logs, (3) attendance and engagement logs, (4) videos, and (5) field notes. Fidelity forms were modified from a previous school-based mindfulness study [76] and included fidelity to curriculum components and core process quality (eg, the day’s planned activities, maintaining engagement, and overall quality). Teacher activity logs documented instances when teachers implemented program activities outside the designated implementation period, including details on which activities were used (eg, weekly primary and booster sessions), how often they were implemented, the duration, and the context in which they occurred. Attendance and engagement logs tracked attendance of children and teachers, as well as engagement, disruptiveness, and open-ended notes per session. Finally, field notes were written per session to document the contextual factors (eg, special considerations for child life events, school disruptions, and technology difficulties).

#### Data Analysis

Qualitative, quantitative, and mixed methods analyses will be used. Scale and subscale reliability was assessed using Cronbach α values. Quantitative data will be analyzed using descriptive and inferential statistics. Standard descriptive statistics are conducted to understand variable distributions (eg, sociodemographic characteristics). Inferential parametric and nonparametric statistics (mean comparisons via 1-tailed *t* tests and linear regression) will be used to answer research questions about program and implementation effects for objectives 2 and 3. Content and thematic analysis approaches will be used to analyze qualitative data (interviews, focus groups, and open-ended survey responses) for objective 1. Qualitative and quantitative findings will be integrated, where appropriate.

### Ethical Considerations

All procedures performed in studies involving human participants were in accordance with the ethical standards of the institutional or national research committee and with the 1964 Helsinki Declaration and its later amendments or comparable ethical standards. The study was approved by the University of Illinois Urbana-Champaign Institutional Review Board (IRB23-0310). All data have been deidentified with unique numeric study IDs created for each participant. Digital copies of data are stored on university-maintained secure servers and password-protected computers. Hard copies of data are stored in locked file cabinets in a secure office. Pseudonyms will be used for qualitative data reporting. Audio files, video files, and photos will be destroyed at the end of the study.

Informed consent for children was obtained from legal guardians, and teachers provided consent for their own participation. Participants had the option to provide consent in person or electronically via the Qualtrics survey platform.

Participants were compensated US $5 for each survey they completed. Those who consented to hair collection and wearable sensors received US $10 for each sample. Parents who consented to provide existing school and observational data received US $10. A bonus of US $15 was given to teachers and parents who provided all data at each time point. Participants who completed interviews or focus groups received US $15. The maximum total for parents and teachers was US $100. Teachers who completed the entire study received a US $150 account transfer to purchase supplies for their classrooms.

## Results

### Demographics and Study Flow of Participants

The intervention and data collection have been completed. Currently, data are being processed for analysis. To determine whether there were group differences in demographic variables between the intervention and waitlist control groups, we conducted *t* tests for continuous variables and chi-square tests (Fisher exact test) for categorical variables. The intervention group was significantly older than the control group (t_30_=−2.97; *P*=.006). There were no other statistical differences in any demographic characteristics.

**Figure 2:**
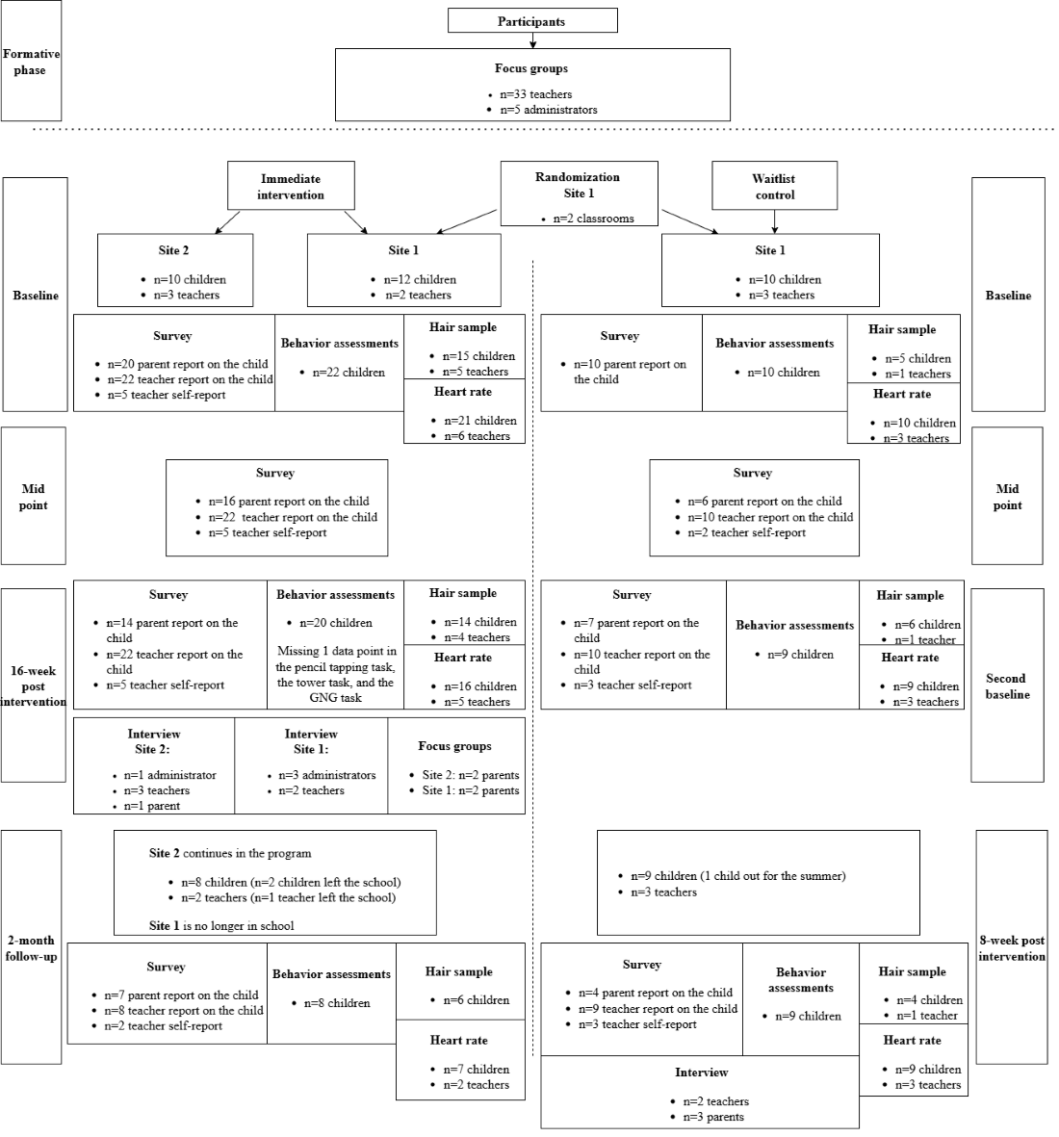
Diagram of the flow of participants through each stage of the study. GNG: go/no-go task.

Overall, there was limited attrition across the study. The study flow and sample sizes for each component are illustrated in Figure 2.

### Feasibility and Acceptability

Although benchmarks or thresholds of success are not agreed upon for pilot studies, we aimed for 75% parent consent for all data collection components. This threshold was achieved for children who consented to wear sensors at >95% and nearly achieved for all research components and hair at >65%. This is consistent with recommendations by Teresi et al [32]. All participating teachers consented to all parts of the research study. Focus groups and interviews revealed acceptability among teachers and administrators who reported that a mindfulness-based intervention in the classroom would be welcome, with a few teachers noting that physiological data collection would require intentional communication with parents who may have reservations; those recommendations were incorporated into recruitment and consenting procedures, although direct quantification remains challenging.

### Scale and Subscale Reliability

Reliability was calculated for each scale and subscale. Cronbach α values for all scales and subscales are presented in Table 5. In total, 26 scales and subscales had Cronbach α values of ≥0.70, 2 scales had values between 0.60 and 0.69, and 5 scales had a value at 0.60. A cutoff value of 0.60 is considered acceptable for reliability [79]. For the 5 subscales with reliability <0.60, we assessed whether removing any items would improve the reliability and found that it increased to >0.60 for 4 of them (Table 5).

**Table 5:** Reliability values for survey measurements.

Measures				Cronbach α^a^		Items, n		Improvement		Items deleted
**Teacher report on child**										
	**Preschool Expulsion Risk Measure**									
		Full scale	0.96		12		—^b^		—	
		Classroom disruption	0.96		3		—		—	
		Fear of accountability	0.83		3		—		—	
		Hopelessness	0.89		3		—		—	
		Teacher stress	0.97		3		—		—	
	**Student-Teacher Relationship Scale**									
		Closeness	0.84		8		—		—	
		Conflict	0.86		7		—		—	
	**Children's Behavior Questionnaire**									
		Surgency	0.82		12		—		—	
		Negative affect	0.57		12		0.61		26^c^	
		Effort control	0.70		12		—		—	
	**Strengths and Difficulties Questionnaire**									
		Hyperactivity	0.89		5		—		—	
		Emotional symptoms	0.68		5		—		—	
		Conduct problems	0.70		5		—		—	
		Peer problems	0.48		5		0.64		23	
		Prosocial behavior	0.90		5		—		—	
		Total difficulties	0.87		20		—		—	
**Teacher report on self**										
	**Perceived Stress Scale**									
		Short	0.70		4		—		—	
		Sum	0.89		10		—		—	
	**Mindfulness in Teaching Scale**									
		Intrapersonal teacher mindfulness	0.59		9		0.71		4^c^	
		Interpersonal teacher mindfulness	0.60		5		—		—	
	**Teacher Social-Emotional Learning Beliefs Scale**									
		Comfort	0.82		4		—		—	
		Commitment	0.94		4		—		—	
		Culture	0.41		4		0.67		12	
**Parent report on child**										
	**Children's Behavior Questionnaire**									
		Surgency	0.77		12		—		—	
		Negative affect	0.80		12		—		—	
		Effort control	0.75		12		—		—	
	**Strengths and Difficulties Questionnaire**									
		Hyperactivity	0.742		5		—		—	
		Emotional symptoms	0.49		5		0.53		16	
		Conduct problems	0.70		5		—		—	
		Peer problems	0.63		5		—		—	
		Prosocial behavior	0.84		5		—		—	
		Total difficulties	0.81		20		—		—	
**Parent report on self**										
	**Perceived Stress Scale**									
		Short	0.79		4		—		—	
		Sum	0.93		10		—		—	

^a^Cronbach α values reported for the preintervention time point only.

^b^Not applicable.

^c^Reverse-coded items.

## Discussion

### Program Outcomes

The study investigates the effect of an MMP on preschool children’s social-emotional, behavioral, and cognitive outcomes, including effects on acute and chronic stress. A mixed methods approach was used, integrating quantitative, qualitative, and physiological data. The study addresses several notable gaps in the literature, including determining MMP effects on a sociodemographically diverse population of preschoolers, identifying differential responses by program component (eg, movement alone and movement plus breathing), and exploring underlying physiological and social-emotional mechanisms of intervention responsivity. Taking this approach will further enrich our understanding of the potential mechanisms and efficacy of mindfulness-based interventions.

### Feasibility and Acceptability of the MMP

Unique to this study is the collection of formative qualitative data used to improve the feasibility and acceptability of programming and the design of the research protocol. Results from this pilot study will inform the scaling of the MMP across multiple sites and settings. This MMP will be improved through our evaluation of program feasibility, including whether the program was implemented with fidelity or adaptations at each site by the program implementers and teachers. Qualitative findings related to program implementation; expectations; and outcomes from parents, teachers, and school administrators will guide modifications. We will use observed engagement levels among children, teachers, and parents to identify any additional improvements to the MMP. Furthermore, the study will determine the feasibility of collecting diverse forms of data in preschool settings, including physiological (heart rate sensor data and hair samples), behavioral, and survey data.

### Strengths

Strengths of the study include its focus on the crucial early childhood period for emotion regulation and behavioral intervention, the investigation of physiological and behavioral mechanisms undergirding the effects of mindfulness on outcomes, and the exploration of program implementation feasibility and acceptability among diverse early childhood populations. The study uses multiple reporters and data sources to triangulate findings and uses a whole-person approach. Moreover, it examines short-term and sustained effects of programming on teacher and child outcomes. It captures potential dose effects by administering 16 weeks of programming to the intervention group and 8 weeks of programming to the waitlist control group. Finally, it incorporates a train-the-teacher model of implementation that can be tested with follow-up, even for 2 months, to inform the need and frequency for boosters and technical assistance.

### Limitations

Limitations include the small sample size of 32 children and 8 teachers and the relatively short follow-up period of only 2 months, leaving the sustained effects uncertain. Teacher effects may affect findings. The use of multiple reporters and data sources (eg, parents, observations, and behavioral tasks) helps reduce potential bias. Finally, some items on subscales had to be deleted to achieve a Cohen κ of at least 0.6, which reduces the number of items.

### Conclusions

Results from this study will enrich our understanding of mindfulness-based interventions on preschool children. Findings from this pilot study will also guide the scaling of this RCT across additional diverse preschool populations. An adequately powered full-scale RCT will provide a better understanding of mechanisms of change and child responsiveness to different MMP components, helping to inform the tailoring of future programs to determine what works best for whom and under which circumstances.

## Abbreviations

DHEA: dehydroepiandrosterone
HPA: hypothalamic-pituitary-adrenal
HPG: hypothalamic-pituitary-gonadal
HRV: heart rate variability
MMP: mindful movement practices
M2M-CC: Mission2Move and Calm Caterpillar
SEL: social-emotional learning
RCT: randomized controlled trial
PBIS: positive behavioral intervention and supports

## Data Availability

The datasets generated or analyzed during this study are not publicly available due to privacy or ethical restrictions.
